# The Diagnosis and Treatment of Acute Cholecystitis: A Comprehensive Narrative Review for a Practical Approach

**DOI:** 10.3390/jcm13092695

**Published:** 2024-05-03

**Authors:** Lara Mencarini, Amanda Vestito, Rocco Maurizio Zagari, Marco Montagnani

**Affiliations:** 1 Department of Medical and Surgical Sciences, University of Bologna, Via Massarenti 9, 40138 Bologna, Italy; lara.mencarini@studio.unibo.it (L.M.); roccomaurizio.zagari@unibo.it (R.M.Z.); 2 Gastroenterology Unit, IRCCS Azienda Ospedaliero-Universitaria di Bologna, 40138 Bologna, Italy; amanda.vestito@aosp.bo.it; 3 Esophagus and Stomach Organic Diseases Unit, IRCCS Azienda Ospedaliero-Universitaria di Bologna, 40138 Bologna, Italy

**Keywords:** acute cholecystitis, early laparoscopic cholecystectomy, cholangitis, pancreatitis, choledocolithiasis, cholecystostomy, EUS gallbladder drainage, cholecystitis in pregnancy, cholecystitis in the elderly, cholecystitis in cirrhosis

## Abstract

Acute cholecystitis (AC), generally associated with the presence of gallstones, is a relatively frequent disease that can lead to serious complications. For these reasons, AC warrants prompt clinical diagnosis and management. There is general agreement in terms of considering early laparoscopic cholecystectomy (ELC) to be the best treatment for AC. The optimal timeframe to perform ELC is within 72 h from diagnosis, with a possible extension of up to 7–10 days from symptom onset. In the first hours or days after hospital admission, before an ELC procedure, the patient’s medical management comprises fasting, intravenous fluid infusion, antimicrobial therapy, and possible administration of analgesics. Additionally, concomitant conditions such as choledocholithiasis, cholangitis, biliary pancreatitis, or systemic complications must be recognized and adequately treated. The importance of ELC is related to the frequent recurrence of symptoms and complications of gallstone disease in the interval period between the onset of AC and surgical intervention. In patients who are not eligible for ELC, it is suggested to delay surgery at least 6 weeks after the clinical presentation. Critically ill patients, who are unfit for surgery, may require rescue treatments, such as percutaneous or endoscopic gallbladder drainage (GBD). A particular treatment approach should be applied to special populations such as pregnant women, cirrhotic, and elderly patients. In this review, we provide a practical diagnostic and therapeutic approach to AC, even in specific clinical situations, based on evidence from the literature.

## 1. Introduction

AC, defined as acute inflammation of the gallbladder wall, is generally related to the presence of gallstones in the cystic duct or in the gallbladder neck. In a minority of cases, AC occurs in the absence of gallstones, such as in cases of predisposing conditions. The importance of AC is related to its frequency and to its clinical impact, requiring adequate management. The mainstay of AC diagnosis and treatment has been delineated in recent expert guidelines [1,2,3,4,5,6]. After clinical, laboratory, and imaging examination, the diagnosis of AC is relatively straightforward. In the course of AC, concomitant gallstone-related complications may occur, such as choledocholithiasis, acute cholangitis, and biliary pancreatitis. In such cases, additional diagnostic workup and adequate therapeutic procedures must be performed.

Based on the available studies, the medical and surgical treatment of AC should follow a defined roadmap. The first therapeutic measures consist of fasting, fluid intravenous infusion, and antimicrobial therapy. Furthermore, analgesics should be administered if needed. In the context of AC, patients should be stratified according to the severity of the clinical features, which only rarely contraindicates ELC. A specific severity grading for AC has been formulated in order to correctly identify patients who are unfit for surgery [1,2]. ELC refers to laparoscopic cholecystectomy, performed within 72 h after hospital admission, or up to 7–10 days from symptom onset [2,6]. ELC is particularly important because it allows for same-admission treatment and cure of both AC and other gallstone-related complications. In fact, ELC minimizes the chance of recurring complications of underlying gallstone disease, such as biliary colic, recurrent cholecystitis, cholangitis, pancreatitis, and their systemic complications.

Because gallstone disease is frequent and increases with age, it is not unusual to treat patients with age-related comorbidities requiring a conservative approach and, possibly, a delayed surgical intervention. In this setting, percutaneous or endoscopic GBD procedures can be performed in order to control the source of infection and to improve the patient’s conditions. Conversely, in case of delayed laparoscopic cholecystectomy (DLC), a 6-week interval is generally recommended.

Another important issue is cholecystectomy in AC patients with concomitant gallstone complications, such as biliary pancreatitis and common bile duct stones. In special groups, namely, pregnant women, patients with liver cirrhosis, and elderly patients, the best risk–benefit ratio for cholecystectomy indication and timing has been evaluated.

The aim of the present review is to provide a practical diagnostic and therapeutic approach to AC for clinicians based on expert guidelines and also including recent studies referring to specific clinical contexts.

## 2. Epidemiology

AC is generally associated with the presence of gallstones, accounting for approximately 90% of all cases, with the remaining 10% being represented by acalculous AC [7,8].

AC represents a common diagnosis at hospital admission, occurring in approximately 3–10% of all patients presenting with abdominal pain at the emergency room [8]. AC mainly affects the elderly adult population, with an increasing incidence in people over the age of 50, and presents a high morbidity rate. The overall AC-related mortality is about 3% and increases in the elderly, particularly in cases of comorbidities [8,9,10]. Differently, a higher rate of mortality occurs in acalculous AC, where it can be as high as 15–40% [8,10,11].

## 3. Etiology

Calculous AC is the most frequent complication of gallstone disease, occurring in approximately 10% of patients with symptomatic gallstones over a ten-year follow-up period [12]. The key event underlying calculous AC is the obstruction of the cystic duct by stones or sludge. The resulting increase in gallbladder intraluminal pressure generates an acute inflammatory response of the gallbladder wall [7]. Sometimes, secondary biliary infection from enteric organisms may occur, most frequently *Escherichia coli*, followed by *Klebsiella*, *Enterococcus,* and *Enterobacter* [4,13]. Well-established risk factors for gallstone disease are obesity, rapid and substantial weight loss [14], female sex, Hispanic and American Indian ancestry [15], medications (e.g., octreotide and ceftriaxone) [16,17], diabetes [18], pregnancy [19], and gastrectomy [20]. In contrast, calculous AC in children is mainly related to congenital disorders (e.g., hemolytic anemia and cystic fibrosis) [21].

In most patients, acalculous AC presents a multifactorial pathogenesis, resulting in stasis and ischemia of the gallbladder wall, with a subsequent local inflammatory response. Well-known risk factors for acalculous AC are sepsis, hypotension, cardiovascular disease, total parenteral nutrition, immunosuppression, major trauma, or burns, typically with a long stay in the intensive care unit [7]. Opportunistic pathogens such as *Cryptosporidium*, Cytomegalovirus, or Microsporidia can sustain acalculous AC in patients with AIDS or in otherwise immunosuppressed patients [22,23]. Acalculous AC can also occur in cases of cystic duct obstruction that is secondary to biliary cancer, extrinsic inflammation, lymphadenopathy, or metastasis [24]. Importantly, acalculous AC is the most frequent form of AC in the pediatric population [25]. In particular, it generally occurs in cases of infectious diseases (e.g., Epstein–Barr virus and hepatitis A virus infection) or parasitosis, systemic vasculitis (e.g., Kawasaki disease and polyarteritis nodosa), and gallbladder or biliary tract congenital malformations [26,27]. In recent years, with the growing burden of obesity in children and adolescents, cholesterol gallstones have become more frequent in the pediatric population [28].

## 4. Diagnosis

The diagnosis of AC is based on clinical presentation, a physical examination, laboratory findings, and an imaging study [1].

### 4.1. Clinical Presentation and Physical Examination

AC should be suspected in patients presenting with right upper quadrant pain, sometimes accompanied by fever, nausea, and vomiting [7]. On physical examination, the presence of a positive Murphy sign (arrest of inspiration during palpation of the right upper quadrant) is very suggestive of AC, with a specificity of 87% to 97% [29,30]. Clinicians can also observe tenderness, pain, or a palpable mass in the right upper quadrant [1]. Jaundice is not typical for AC and may suggest severe AC with common bile duct stones, with or without concurrent acute cholangitis [1,31].

### 4.2. Laboratory Tests

In the course of AC, the main laboratory findings are leukocytosis and increased C-reactive protein [1]. A marked increase in bilirubin and hepatobiliary enzymes may indicate concomitant choledocholithiasis, and possibly acute cholangitis [31]. Furthermore, acute hepatitis must be ruled out. For this purpose, using clinical and imaging findings can assist in the correct diagnosis of AC [32]. The overall usefulness of procalcitonin for the diagnosis of sepsis has been debated [33]. On the other hand, procalcitonin levels have been found to be associated with AC severity [1,33,34].

### 4.3. Imaging Findings

Ultrasound (US) is the most employed imaging technique for the initial diagnosis of AC. Thickening of the gallbladder wall (>3 mm) with a layered appearance, gallstones or retained debris, pericholecystic fluid, and gallbladder enlargement are the typical sonographic signs of AC. Furthermore, a positive sonographic Murphy sign (tenderness elicited by the compression of the transducer over the gallbladder) can be observed [1].

In clinical practice, US can be performed directly at the patient’s bedside, at the doctor’s office, or in the emergency department. In particular, point-of-care ultrasound (POCUS) is an important approach for real-time imaging support in the course of clinical evaluation. Furthermore, US can easily be repeated in AC patients, who require monitoring over time [35].

**Figure 1:**
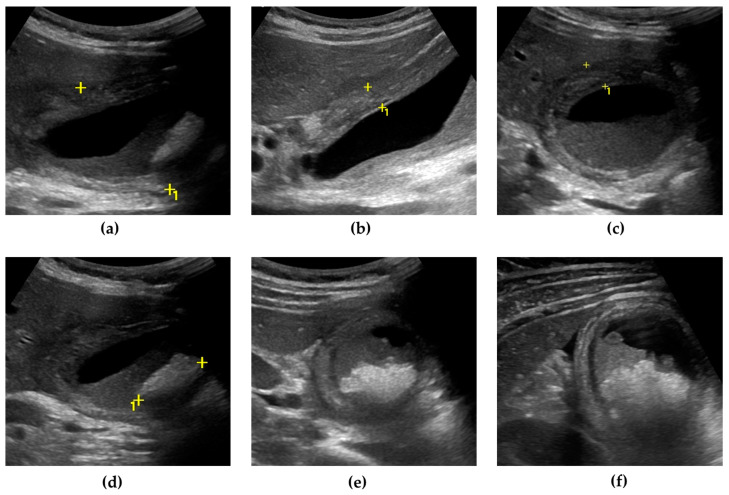
Gangrenous cholecystitis. The gallbladder is markedly distended, with an antero-posterior diameter greater than 5 cm (calipers) (**a**). The gallbladder walls are thickened (up to 10 mm), with a layered appearance, showing multiple striations and alternating hypo/hyperechoic bands (calipers) (**b**,**c**). Inside the gallbladder lumen, a significant amount of biliary sludge (non-shadowing echoic material, determining a horizontal fluid–fluid level) surrounds a microlithiasis aggregate, a brighter echoic material with an acoustic posterior shadow (calipers) (**d**,**e**). A small triangular fluid collection is present between the gallbladder and liver surface (**f**).

**Figure 2:**
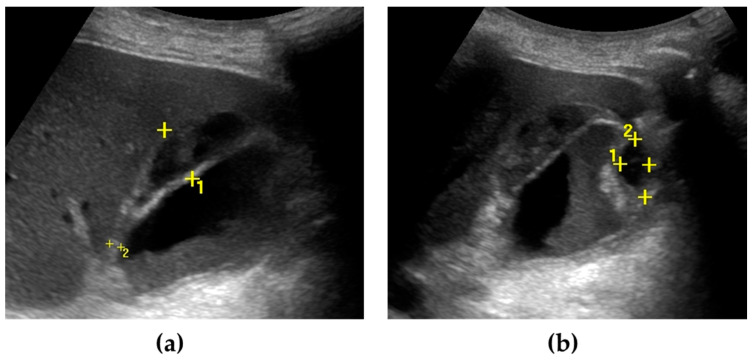
Gallbladder perforation. The gallbladder is distended, with irregular thickening of the walls. Multiple pericholecystic collections are shown (calipers) (**a**,**b**). Biliary sludge can be seen within the gallbladder lumen (**a**).

US can also detect AC complications. Gangrenous cholecystitis (Figure 1) is characterized by a thickened and irregular gallbladder wall, sometimes with desquamated mucosa, appearing as an intraluminal flap [36,37]. A defect of the gallbladder wall (“hole sign”) represents the direct visualization of parietal perforation (Figure 2), often communicating with pericholecystic collections or surrounded by hyperechoic mesenteric reactions [37,38]. Additionally, US can be useful in differentiating gallbladder empyema, emphysematous cholecystitis, and a phlegmonous reaction or pericholecystic abscesses [1,39,40].

Second-level imaging techniques (CT and MRI) are indicated in case of a doubtful diagnosis or to confirm suspected complications of AC. In particular, CT is the technique of choice for the diagnosis of emphysematous cholecystitis, because it allows for the detection of minute gas bubbles, which appear as hypodense spots [1,41]. Magnetic resonance cholangiopancreatography (MRCP) is useful for evaluating concurrent choledocholithiasis or alterations of the biliary tract [42,43].

Hepatobiliary scintigraphy (HIDA scan) is the most sensitive and specific test for AC, which is associated with the absence of radiotracer uptake in the gallbladder before and after morphine administration. However, a HIDA scan is a long-duration procedure and involves exposure to radionuclides [7,44].

Recently, contrast-enhanced US (CEUS) has proven to be useful to detect gallbladder perforation and to characterize pericholecystic abscesses [45,46].

## 5. Clinical Evolution

AC is an acute inflammatory disease of the gallbladder that sometimes can progress to a number of local complications, such as gangrenous cholecystitis, gallbladder perforation, pericholecystic abscess, biliary peritonitis, biliary fistula, emphysematous cholecystitis, gallbladder empyema, and hemorrhagic cholecystitis [1]. In a minority of cases, systemic complications may occur.

- Gangrenous cholecystitis. Transmural inflammation and ischemic necrosis of the gallbladder wall, occurring approximately in 20% of cases, is the most common complication of AC [37].
- Emphysematous cholecystitis. This is characterized by intraluminal or intramural proliferation of gas-forming organisms (e.g., *Klebsiella*, *Clostridium*, or *Escherichia coli*) [37].
- Gallbladder empyema (suppurative cholecystitis). This complication develops when purulent material accumulates within a distended gallbladder in the course of AC, which is due to a persistent obstruction of the cystic duct and bile stasis, with bacterial proliferation [47,48].
- Gallbladder perforation. This occurs in about 10% of patients with AC and consists of a loss of continuity of the gallbladder wall, mainly due to ischemia and necrosis, generally located in the fundus of the organ. In most cases, it is a covered perforation, delimited by the surrounding tissue [8].
- Biliary peritonitis. Rarely, free perforation into the peritoneum can occur. The consequent bile leakage in the peritoneal cavity leads to biliary peritonitis, a condition associated with high mortality [8].
- Pericholecystic and hepatic abscess. Gallbladder perforation can evolve into a pericholecystic or even hepatic abscess, which is due to the spread of bacterial infection [1].
- Cholecystoenteric fistula. This is an uncommon complication of gallstone disease, characterized by a fistula between the gallbladder and the gastrointestinal tract, mainly with the duodenum, rarely with the colon, and exceptionally with different gastrointestinal segments [49].
- Mirizzi syndrome. A stone impacted in the cystic duct or in the gallbladder neck can determine a common hepatic duct obstruction by means of extrinsic compression, with consequent cholestasis. In this setting, a biliary fistula may develop between the gallbladder and the common bile duct (cholecystocholedochal fistula) [49].
- Gallstone ileus and Bouveret syndrome. Very rarely, gallstones may pass through a cholecystoenteric fistula and, if more than 2.5 cm in size, they can impact the terminal ileum at the level of the ileocecal valve, leading to mechanical bowel obstruction (gallstone ileus). Exceptionally, the gallstone impacts in the duodenum, causing a gastric outlet obstruction (Bouveret syndrome) [49].
- Hemorrhagic cholecystitis. The presence of blood inside the gallbladder lumen is mainly due to the rupture of a hepatic artery pseudoaneurism. Traditionally, the clinical presentation consists of Quinckle’s triad (biliary colic, jaundice, and overt upper gastrointestinal bleeding) [36].

According to the Tokyo guidelines, AC can be classified into grade I (mild), grade II (moderate), and grade III (severe). Mild AC represents a disease confined to the gallbladder, in the absence of local and/or systemic complications. Differently, moderate AC develops when at least one of the aforementioned local complications occurs, mainly gangrenous cholecystitis, pericholecystic abscess, biliary peritonitis, or emphysematous cholecystitis. An elevated WBC count (>18.000/mm^3^), a palpable tender mass in the right upper abdominal quadrant, and a duration of symptoms greater than 72 h are also associated with moderate AC. Severe AC occurs when the disease leads to systemic complications, with at least one organ failure (cardiovascular, neurological, respiratory, renal, hepatic, or hematological dysfunction) [1].

## 6. Treatment

The treatment of AC is based on the disease severity, the presence of complications, and pre-existing conditions and comorbidities. ELC represents the cornerstone in the treatment of AC, but, in some circumstances, when ELC is contraindicated, delayed surgery is performed. Medical treatment, in particular antibiotic therapy, is also of pivotal importance. Sometimes, GBD placement may be indicated [2].

### 6.1. Medical Treatment

In the course of AC, clinicians should keep the patient on fasting and initiate antimicrobial therapy. General supportive care, such as fluid and electrolyte intravenous infusion, and possibly analgesic agent administration, are also mandatory [3].

In order to select a suitable empirical treatment, generally based on broad-spectrum antibiotics (e.g., penicillin, cephalosporins, fluoroquinolones), clinicians should consider drug pharmacokinetics and pharmacodynamics, local antibiogram, a history of antimicrobial use, allergic or adverse reactions, and renal and hepatic function. Importantly, the presence of a biliary–enteric anastomosis warrants anaerobic therapy (e.g., metronidazole) [50]. Severe and healthcare-associated infections can be sustained by Pseudomonas species; therefore, in such cases, antimicrobial therapy against this pathogen is recommended [51].

Blood and, possibly, bile cultures are requested for all stages of AC, except for the mild form of the disease, if they are community-acquired. Of note, a culture of bile and gallbladder tissue is suggested during cholecystectomy in case of emphysematous cholecystitis, gallbladder wall necrosis, or perforation [4]. Once cultures and susceptibility test results are available, clinicians should discontinue antimicrobial therapy if no longer needed or switch to an antimicrobial agent that is specific for the isolated organism (antimicrobial de-escalation) [52].

The duration of antibiotic therapy depends on clinical features. In patients with mild or moderate AC who are candidates for ELC, antimicrobial therapy is recommended from the diagnosis until surgical intervention or further, if clinically indicated [53,54]. Particular attention should be paid to patients at a high risk of bacterial infection or antimicrobial resistance, as in the case of immunosuppression therapy or healthcare-associated infections [6]. Diabetes is also considered a risk factor for the failure of conservative management [55]. In patients with severe AC, antibiotic treatment should be further extended for 4–7 days after the source of infection is controlled. In case of local complications such as pericholecystic abscesses or gallbladder perforation, the antimicrobial therapy should be discontinued only when the local, systemic, and laboratory (e.g., procalcitonin serum level) signs of infection have disappeared [4].

### 6.2. Diagnosis and Treatment of Gallstone-Associated Disease

The presence of common bile duct stones is reported in about 5% to 15% of patients with calculous AC. A prompt recognition of this condition is of relevance in clinical practice, because diagnosis and management of choledocholithiasis by endoscopic retrograde cholangiopancreatography (ERCP) is a priority.

As discussed above, the raising of serum hepatobiliary markers, mainly bilirubin, is associated with choledocholithiasis and, in the appropriate clinical setting, it suggests a concomitant acute cholangitis.

Besides diagnosing AC, abnormalities of the biliary tree can be detected by US, from bile duct enlargement to direct visualization of stones in the lumen of the common bile duct, the latter requiring therapeutic ERCP. Notably, Mirizzi syndrome can be mistaken for choledocholithiasis [6].

Predictive factors for common bile duct stones have been evaluated. A common bile duct diameter > 6 mm (with the gallbladder in situ), total serum bilirubin level > 1.8 mg/dL, abnormal liver biochemical test other than bilirubin, age older than 55 years, and clinical gallstone pancreatitis are reported to be associated with choledocholithiasis in 10% to 50% of cases. The moderate risk related to these conditions justifies a second-level imaging in order to detect patients who need therapeutic ERCP. According to local expertise, a detailed evaluation of the biliary tree can be performed preoperatively by EUS or MRCP, or intraoperatively using laparoscopic US or cholangiography.

In the absence of the above factors, the risk of concomitant bile duct stones is so low (<10%) that ELC can be performed without further investigation [6,56].

Therefore, having access to EUS and MRCP in the short term may conditionate the timing of cholecystectomy.

### 6.3. Surgery (Cholecystectomy)

**Figure 3:**
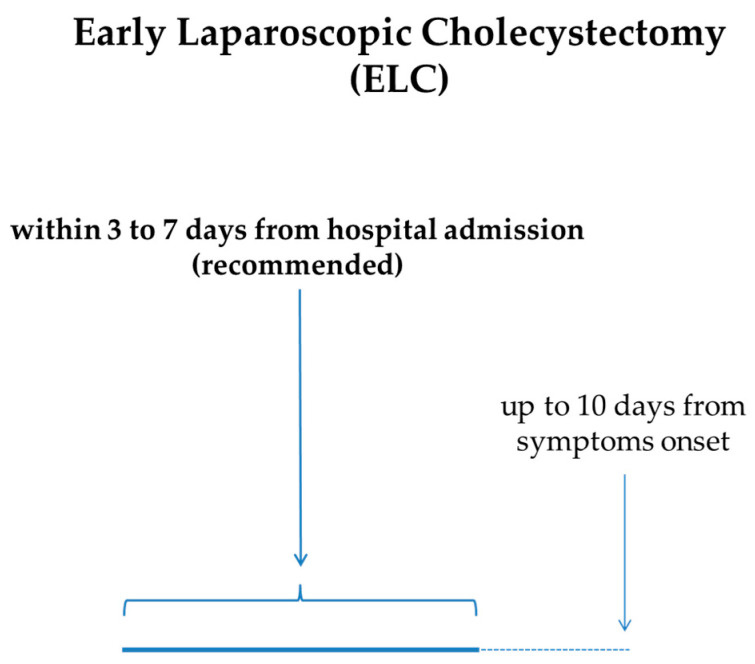
Recommended timeframe for ELC in AC from onset of symptoms and/or hospital admission.

The cornerstone of AC treatment is ELC. In particular, ELC performed within 72 h should be the method of choice for the treatment of AC, because it is related to a shorter hospital stay, fewer perioperative complications, and reduced costs [57,58,59]. The quality of the evidence for this statement is considered to be moderate, and the strength of recommendation is strong. Furthermore, a 7- to 10-day timeframe from the clinical onset of AC to ELC is now considered acceptable [6]. Altogether, the expert guidelines recommend very early (≤72 h from symptom onset) or early (<7–10 days from symptom onset) laparoscopic cholecystectomy, even if high-quality definitive evidence is lacking. In cases in which ELC cannot be performed, DLC can be planned. There is a temporal frame, ranging from 1 to 6 weeks after the onset of AC, in which laparoscopic cholecystectomy is not recommended because of a common concern of an increased risk of serious adverse events [6]. Therefore, even if the level of evidence is very low and the strength of recommendation is weak, for patients who cannot undergo ELC within 7 [2] or 10 days [6] from symptom onset, it is suggested to delay surgery beyond 6 weeks (Figure 3).

In patients with mild AC but with a concomitant high surgical risk, ELC can be performed once the medical treatment has improved the patient’s general condition [2].

In contrast, in patients with moderate AC, ELC must be preceded by medical therapy because of the possible surgical challenges related to the inflammatory reaction [2].

In cases of severe AC, ELC should be performed only with the availability of intensive care support and in patients with factors that are predictive of clinical recovery. For example, early remission of cardiovascular or renal failure after admission is considered a favorable organic systemic failure (FOSF). According to the Tokyo guidelines, a bilirubin serum level ≥2 mg/dL, as well as neurologic and/or respiratory dysfunction, are considered negative predictive factors that contraindicate ELC in patients with grade III AC. Furthermore, it is of primary importance to evaluate the performance status in patients who are candidates for early surgery. Indeed, patients affected by severe AC with a Charlston Comorbidity Index (CCI) greater than 4 and/or American Society of Anesthesiologists physical status classification score (ASA-PS) above 3 are considered at high risk for surgery [2]. In particular, according to CCI, the presence of a metastatic solid tumor or acquired immunodeficiency syndrome (AIDS) is considered such a high-risk condition that it contraindicates ELC in patients with severe AC. Moderate-to-severe liver or renal disease, leukemia, lymphoma, cancer without metastasis, diabetes mellitus with chronic complications, and cerebrovascular (hemiplegia) events are considered moderate risk conditions by themselves. However, in such patients, the presence of an additional comorbidity contraindicates ELC. Particular caution must be observed in patients receiving steroid treatment, immunosuppressive therapy (e.g., transplant recipients), or biological drugs [60,61,62,63]. If laparoscopic cholecystectomy cannot be performed during the primary admission for AC, DLC should be planned after complete clinical recovery and at least 6 weeks after clinical onset [2] (Table 1).

The recurrence of AC represents a relatively frequent clinical scenario, accounting for almost one-quarter of patients treated conservatively during the first episode of AC [64]. Multiple factors can influence the risk of recurrence in such patients. Notably, recurrent AC appears to be more severe than the first episode [65] and to be associated with an increased risk of different biliary diseases, such as obstructive jaundice or gallstone pancreatitis [66]. According to recent evidence, 20% to 38% of patients with AC undergoing percutaneous transhepatic GBD that is not followed by delayed cholecystectomy experience a recurrence of AC, mainly within three months from the index event [67].

Traditionally, there have been concerns about cholecystectomy in specific subgroups of patients, namely, pregnant women, cirrhotic patients, and elderly patients (Table 2).

In pregnant women with AC, the conservative approach is associated with relapse rates in the range of 40–70%. Some concerns have been raised regarding surgery in the first trimester because of the potential risk of miscarriage and toxicity for the fetus related to anesthesia. The optimal time for laparoscopic cholecystectomy is considered the second trimester. Patients in the near term can be managed conservatively in order to postpone surgery until after delivery, considering that in the third trimester, there are some concerns related to the size of the uterus [6,68,69,70,71]. Despite the consensus on performing laparoscopic cholecystectomy preferentially during the second trimester, in selected cases, when justified by a favorable risk–benefit ratio, the surgical intervention can be performed in the first- or third trimester [72].

In patients with liver cirrhosis with a Child–Pugh score of A or B and/or with a Mayo End-stage for Liver Disease (MELD) score of less than 15, laparoscopic cholecystectomy in the course of AC is considered the first therapeutic choice, because the risk of liver decompensation after surgery is still acceptable. On the contrary, cholecystectomy is generally contraindicated in patients with liver cirrhosis with a Child–Pugh score of C or a MELD score higher than 15, in which a conservative approach, such as GBD placement, is suggested [7,73].

In elderly patients, ELC should be considered, even if the patient is 80 years of age or older. In fact, recent evidence shows a comparable perioperative morbidity and mortality to the younger population. Frailty and surgical scores can assist in the therapeutic decision [74,75].

Another special group of patients is represented by those with AC and concomitant mild acute biliary pancreatitis. The only study specifically designed to address this issue [76] demonstrates that ELC is a better strategy with respect to DLC, which is in line with the standard recommendations for AC. In fact, despite a similar surgical complication rate, the group with delayed surgery displayed a significantly higher occurrence of preoperative biliary-related events (biliary pancreatitis, cholangitis, cholecystitis, biliary colic) and a longer hospital stay [76]. Similarly, the World Society of Emergency Surgery (WSES) guidelines recommend laparoscopic cholecystectomy during initial admission for patients with mild acute gallstone pancreatitis, but in this case, the presence of a concomitant AC is not specifically considered [77].

### 6.4. Gallbladder Drainage

GBD, also known as cholecystostomy, should be performed in all patients with severe AC in whom cholecystectomy is contraindicated. Moreover, GBD should also be considered in patients with moderate AC and a high surgical risk, particularly in case of an inadequate response to the medical treatment [2]. Percutaneous transhepatic GBD, performed under US guidance, is the method of choice. In contrast, percutaneous GBD through the transperitoneal route is not recommended, because it is associated with a higher rate of complications, mainly bile leakage and biliary peritonitis [5,78]. Currently, recent guidelines do not provide any recommendations regarding the time of GBD tube removal. Traditionally, the GBD tube is left in place until cholecystectomy. In case of DLC or if a cholecystectomy is not a therapeutic option, the GBD tube should be removed. Recent evidence shows that an early tube removal (about 7–10 days) can be feasible and safe, especially if GBD has been performed by the transhepatic route. Before tube removal, clinicians should verify the disappearance of local and systemic signs of infection, patency of the cystic and bile duct, and absence of peritoneal bile leakage. This can be achieved either by using fluoroscopy or by using intracavitary CEUS, as recently described [79]. The correct positioning of the drainage can also be checked during the B-mode US examination. Some authors suggest performing a clamping test before GBD tube removal [80,81,82,83]. Despite these promising data, the real efficacy, appropriate use, and exact timing of cholecystostomy have been questioned based on a number of studies performed in different clinical settings [84].

In recent years, endoscopic ultrasound-guided GBD (EUS-GBD) has proven to be a good alternative to percutaneous GBD for high-surgical-risk patients [85]. According to this technique, the gallbladder is punctured under EUS guidance from the body or antrum of the stomach or from the duodenal bulb. Successively, a lumen-apposing metal stent (LAMS), connecting the gastrointestinal lumen with the gallbladder lumen, is positioned. Some concerns have been raised about technical difficulties in performing cholecystectomy following EUS-GBD, mainly because of the fistulous tract. More data are needed to provide clearer information on the outcome of this technique. In selected high-risk patients, the LAMS can be left in place, but long-term adverse events are described, e.g., LAMS dislocation or occlusion with food, leading to recurrent AC [86,87]. According to recent evidence, EUS-GBD has the advantage of a decreased rate of adverse events and less need for re-intervention, with a comparable success rate to percutaneous GBD [87,88,89,90]. However, these studies did not discriminate between the transhepatic and transperitoneal route in percutaneous GBD.

An alternative endoscopic approach for GBD is based on ERCP with selective cannulation of the cystic duct and a transpapillary stent placement. In particular, this approach should be preferred in patients requiring ERCP for concurrent choledocholithiasis [5,86,91].

In summary, the optimal drainage method (percutaneous/endoscopic) depends on individual patient characteristics and the individual center’s expertise [5,86,92].

## 7. Conclusions

AC is mainly related to the presence of gallstones, and the burden of these diseases is growing with the increase in life expectancy. The diagnosis of AC is based on the initial clinical suspicion, together with laboratory and imaging findings. In recent years, severity grading scores for AC have been developed in order to select the best therapeutic strategy. The gold standard of surgical treatment is laparoscopic cholecystectomy, preceded by medical therapy. Whenever feasible and in the presence of adequate local expertise, ELC is recommended within 72 h from hospital admission or within a maximum of 7 to 10 days from symptom onset [2,6]. In cases of DLC, a timeframe of at least 6 weeks from symptom onset is suggested. Notably, ELC minimizes the recurrence of symptoms and complications in AC patients, given that cholecystectomy can sometimes be challenging and requires bail-out options [84]. Besides health instances, ELC is also preferable to DLC because of the lower healthcare-related costs. On the basis of recent guidelines, laparoscopic cholecystectomy is also indicated in the elderly, in patients with compensated liver cirrhosis, and in pregnant women, preferably in the second trimester. In high-risk AC patients who are not eligible for ELC, rescue or bridge procedures can be indicated. In particular, percutaneous GBD has been widely employed, while EUS-GBD has been developed recently and will possibly be implemented in clinical practice.

## Figures and Tables

**Table 1:** Management of AC according to Tokyo severity grading.

AC Severity Grading	Clinical Features	Surgical Management
Mild AC	Disease confined to the gallbladder, absence of local and systemic complications. Clinical presentation: right upper quadrant pain (±fever/nausea/vomiting/Murphy sign) Laboratory tests: leukocytosis, increased CRP Imaging studies (US/CT/MR/HIDA scan): gallbladder wall thickening with layered appearance, gallstones/retained debris (±gallbladder enlargement/pericholecystic fluid), absence of radiotracer uptake in the gallbladder	ELC
Moderate AC	Local complications (gangrenous cholecystitis, pericholecystic abscess, biliary peritonitis, emphysematous cholecystitis) and/or WBCs > 18.000/mm^3^ and/or palpable tender mass in the right upper abdominal quadrant and/or duration of symptoms > 72 h	ELC or DLC (in patients not fit for surgery at hospital admission)
Severe AC	Systemic complications with at least one organ failure/dysfunction (cardiovascular, neurological, respiratory, renal, hepatic, or hematological dysfunction)	ELC (only if intensive care support is available) in patients with favorable clinical state or GBD (if ELC is contraindicated) followed by DLC after complete clinical recovery

AC: acute cholecystitis. CRP: C-reaction protein. US: ultrasound. CT: computed tomography. MR: magnetic resonance. HIDA scan: hepatobiliary scintigraphy. ELC: early laparoscopic cholecystectomy. WBCs: white blood cells. DLC: delayed laparoscopic cholecystectomy. GBD: gallbladder drainage.

**Table 2:** Cholecystectomy for AC in special clinical settings.

Clinical Setting	Decision-Making for Cholecystectomy
Pregnant women	- Laparoscopic cholecystectomy should be performed preferentially during the second trimester (ELC or DLC) - Surgical intervention can exceptionally be performed in the first or in the third trimester, if necessary
Liver cirrhosis	- Child–Pugh score of A or B: ELC is the first therapeutic choice, if clinically indicated - Child–Pugh score of C: laparoscopic cholecystectomy is generally not indicated because of the risk of liver decompensation after surgery (conservative management—such as GBD—is suggested)
Elderly patients	ELC should be considered; frailty and surgical scores can assist in the therapeutic decision
Concomitant acute mild biliary pancreatitis	In mild biliary pancreatitis, ELC is a better strategy with respect to DLC
Concomitant choledocholithiasis	According to local expertise, a detailed evaluation of the biliary tree can be performed preoperatively by EUS or MRCP, or intraoperatively using laparoscopic US or cholangiography The presence of common bile duct stones warrants therapeutic ERCP pre-, intra- or post-operatively

ELC: early laparoscopic cholecystectomy. GBD: gallbladder drainage. DLC: delayed laparoscopic cholecystectomy. EUS: endoscopic ultrasound. MRCP: magnetic resonance cholangiopancreatography. US: ultrasound.
